# Anti-Inflammatory and Anti-Oxidative Effects of luteolin-7-*O*-glucuronide in LPS-Stimulated Murine Macrophages through TAK1 Inhibition and Nrf2 Activation

**DOI:** 10.3390/ijms21062007

**Published:** 2020-03-16

**Authors:** Young-Chang Cho, Jiyoung Park, Sayeon Cho

**Affiliations:** 1 College of Pharmacy, Chonnam National University, 77 Yongbong-ro, Buk-gu, Gwangju 61186, Korea; cycjjang@hanmail.net; 2 College of Pharmacy, Chung-Ang University, 84 Heukseok-ro, Dongjak-gu, Seoul 06974, Korea; cynical-0528@hanmail.net

**Keywords:** luteolin-7-*O*-glucuronide, anti-inflammation, anti-oxidation, RAW 264.7 macrophages, transforming growth factor beta-activated kinase 1, nuclear factor-erythroid 2 p45-related factor 2

## Abstract

Various herbal extracts containing luteolin-7-*O*-glucuronide (L7Gn) have been traditionally used to treat inflammatory diseases. However, systemic studies aimed at elucidating the anti-inflammatory and anti-oxidative mechanisms of L7Gn in macrophages are insufficient. Herein, the anti-inflammatory and anti-oxidative effects of L7Gn and their underlying mechanisms of action in macrophages were explored. L7Gn inhibited nitric oxide (NO) production in lipopolysaccharide (LPS)-stimulated RAW 264.7 macrophages by transcriptional regulation of inducible NO synthase (*iNOS*) in a dose-dependent manner. The mRNA expression of inflammatory mediators, including cyclooxygenase-2 (*COX-2*), interleukin-6 (*IL-6*), *IL-1β*, and tumor necrosis factor-α (*TNF-α*), was inhibited by L7Gn treatment. This suppression was mediated through transforming growth factor beta-activated kinase 1 (TAK1) inhibition that leads to reduced activation of nuclear factor-κB (NF-κB), p38, and c-Jun N-terminal kinase (JNK). L7Gn also enhanced the radical scavenging effect and increased the expression of anti-oxidative regulators, including heme oxygenase-1 (HO-1), glutamate-cysteine ligase catalytic subunit (GCLC), and NAD(P)H quinone oxidoreductase 1 (NQO1), by nuclear factor-erythroid 2 p45-related factor 2 (Nrf2) activation. These results indicate that L7Gn exhibits anti-inflammatory and anti-oxidative properties in LPS-stimulated murine macrophages, suggesting that L7Gn may be a suitable candidate to treat severe inflammation and oxidative stress.

## 1. Introduction

Macrophages provide the first line of defense against external pathogens. One of the most potent pathogens for macrophage activation is bacterial lipopolysaccharide (LPS), the toll-like receptor 4 (TLR4) ligand [1,2]. Upon stimulation by LPS, macrophages release various inflammatory mediators, including tumor necrosis factor (TNF)-α, interleukin (IL)-1β, IL-6, nitric oxide (NO), and prostaglandin E_2_ (PGE_2_) [3]. Under normal conditions, these inflammatory mediators protect the human body against infectious diseases. However, excessive inflammatory responses, which result in overproduction of inflammatory mediators, may lead to various inflammatory diseases, such as arthritis, asthma, multiple sclerosis, inflammatory bowel disease, and atherosclerosis [4,5,6].

Overproduction of reactive oxygen species (ROS) caused by oxidative stress is implicated in the pathogenesis of several human diseases, including atherosclerosis, cancer, neurodegenerative diseases, and aging [7,8,9]. Cells protect themselves from ROS by inducing the activities of intracellular phase II enzymes [10]. Among these, heme oxygenase-1 (HO-1) plays a pivotal role in maintaining cellular redox homeostasis to protect cells against oxidative stress [11]. HO-1 protein induction is transcriptionally regulated by an inducible transcription factor, nuclear factor-erythroid 2 p45-related factor 2 (Nrf2). Activated Nrf2 translocates into the nucleus and binds to the antioxidant response element (ARE) enhancer, which induces the expression of anti-oxidative genes, including HO-1, GCLC, and NQO1 [12]. Therefore, Nrf2 is a pivotal transcriptional regulator of anti-oxidative genes.

**Figure 1:**
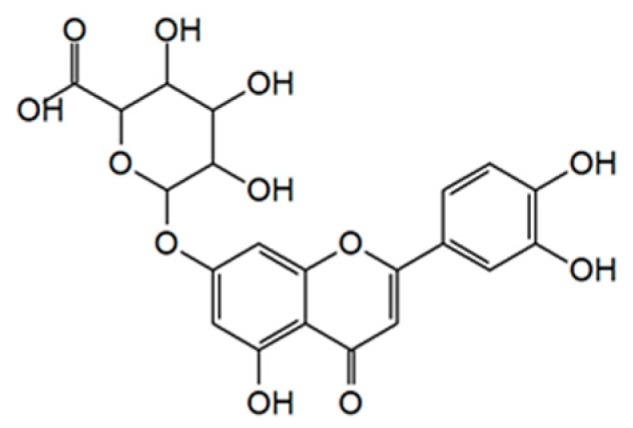
Chemical structure of luteolin-7-*O*-glucuronide (L7Gn).

Pharmacologically effective plant extracts traditionally used to treat diseases share phytochemicals. Luteolin-7-*O*-glucuronide (L7Gn, Figure 1) is a phytochemical common to various anti-inflammatory plant extracts, including those of *Perilla frutescens*, *Remirea maritima*, *Codariocalyx motorius*, and *Ixeris dentata* [13,14,15,16]. Recently, L7Gn was identified as the major constituent of *Cirsium japonicum* CJ-50 fractions responsible for its anti-inflammatory effects in macrophages, elucidated to inhibit c-Jun N-terminal kinase (JNK) for its anti-inflammatory effects [17]. However, the effect of L7Gn on another major inflammatory signaling pathway, the nuclear factor-κB (NF-κB) signaling pathway, and its upstream kinases has not been fully assessed. Previously, it was reported that luteolin, a mother compound of L7Gn, showed anti-inflammatory effects by inhibiting NF-κB signaling pathway and thereby L7Gn-mediated NF-κB regulation could be predicted [18,19]. Furthermore, the anti-oxidative mechanism of action by L7Gn was also insufficient. Therefore, the regulatory mechanism of L7Gn-mediated anti-inflammatory and anti-oxidative effects in RAW 264.7 macrophages were elucidated in this study.

## 2. Results and Discussion

### 2.1. L7Gn Inhibits the Production of Proinflammatory Mediators in RAW 264.7 Macrophages

**Figure 2:**
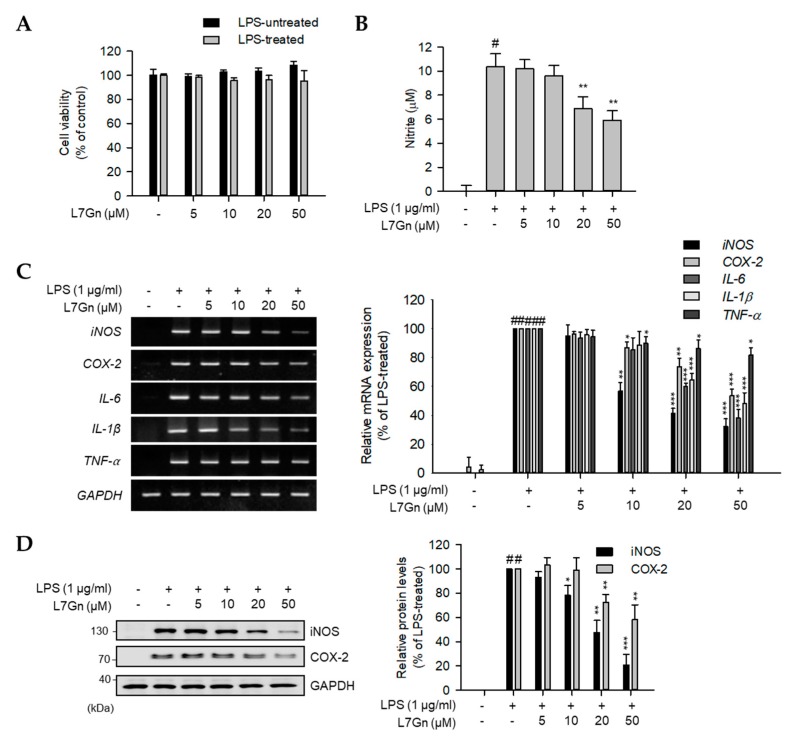
Inhibitory effects of L7Gn on the production of proinflammatory mediators. (**A** and **B**) RAW 264.7 macrophages were pre-treated with various concentrations of L7Gn (0, 5, 10, 20, and 50 μM) for 2 h, and incubated in the presence or absence of lipopolysaccharide (LPS) (1 μg/mL) for an additional 24 h. (**A**) Cell viability was examined using EZ-Cytox assay solution and results were expressed relative to those for the untreated control group. Data represent the mean ± standard deviation (S.D.). (**B**) Nitric oxide (NO) secretion levels in culture media measured using Griess reagent. NO levels were calculated according to a standard curve plotted using a nitrite standard solution. Data represent the mean ± S.D. (**C** and **D**) RAW 264.7 cells were pre-treated with L7Gn (0, 5, 10, 20, and 50 μM) for 2 h and then incubated with LPS (1 µg/mL) for indicated times (3 h for RT-PCR and 24 h for immunoblotting analyses). (**C**) Total RNA was extracted and reverse transcribed to cDNA. Inducible NO synthase (*iNOS*), cyclooxygenase-2 (*COX-2*), and pro-inflammatory cytokines were amplified by PCR and detected on agarose gel. The representative data are shown. (**D**) Total cell lysates were prepared and immunoblot analysis was performed. The protein expression of iNOS and COX-2 was detected using an ECL system and quantified using the LabWorks software. Expression levels of iNOS and COX-2 proteins were normalized to that of GAPDH. Relative expression levels of iNOS and COX-2 are represented as a bar graph (lower panel). Data represent the mean ± S.D. # *p* < 0.01 relative to the LPS-treated and L7Gn-untreated control group. * *p* < 0.05, ** *p* < 0.01, and *** *p* < 0.001 relative to the LPS-treated and 5 μM L7Gn-treated group.

Prior to the elucidation of the biological effects of L7Gn in RAW 264.7 macrophages, the cytotoxic effects of L7Gn were evaluated using a cell viability assay. Since no cytotoxicity was observed upon treatment of the cells with L7Gn (5–50 µM) (Figure 2A), L7Gn concentrations below 50 µM were used for further investigation.

The anti-inflammatory and anti-oxidative effects of L7Gn were evaluated by measuring its inhibitory effect on NO production in LPS-stimulated RAW 264.7 macrophages. As expected, L7Gn showed an inhibitory effect on LPS-induced NO production (Figure 2B). L7Gn-mediated inhibition of NO production was caused by the inhibition of inducible NO synthase (iNOS) mRNA and protein expression (Figure 2C,D). Since the transcriptional inhibition of *iNOS* is caused by the inhibition of the major inflammatory signaling pathways such as NF-κB, mitogen-activated protein kinase (MAPK), and the activation of HO-1 via the Nrf2 pathway, it was hypothesized that L7Gn might play regulatory roles in these signaling pathways.

To elucidate anti-inflammatory effects of L7Gn and their underlying mechanism of action, the mRNA expression levels of various inflammatory mediators, including cyclooxygenase-2 (*COX-2*), *IL-1β*, *IL-6*, and *TNF-α*, in LPS-stimulated RAW 264.7 macrophages were measured (Figure 2C). LPS-induced mRNA expression levels of *COX-2*, *IL-6*, and *IL-1β* were clearly reduced by L7Gn treatment, whereas *TNF-α* mRNA levels were moderately reduced. Furthermore, the COX-2 protein expression was also inhibited in LPS-stimulated RAW 264.7 cells upon treatment with L7Gn (Figure 2D). These data imply that L7Gn might exert anti-inflammatory effects by the transcriptional regulation of inflammatory-mediator expression in macrophages.

Based on the data in Figure 2, L7Gn-mediated inhibition of the cytokine expression levels was more obvious for *IL-6* and *IL-1β* than that of *TNF-α*. This could be addressed by the fact that *IL-6*, *IL-1β*, and *TNF-α* have different binding sites for transcription factors in the promoter regions. Previous studies have shown that *IL-6* and *IL-1β* gene promoters have NF-κB, AP-1, and STAT-binding domains, whereas the *TNF-α* promoter has NF-κB and AP-1-binding regions but does not possess a STAT-binding region [20,21]. These imply that L7Gn might be involved in the regulation of the STAT signaling pathway, which in turn regulates the expression of *IL-6* and *IL-1β* genes in LPS-stimulated macrophages.

### 2.2. L7Gn Alleviates NF-κB, p38, and JNK Activation in RAW 264.7 Macrophages

**Figure 3:**
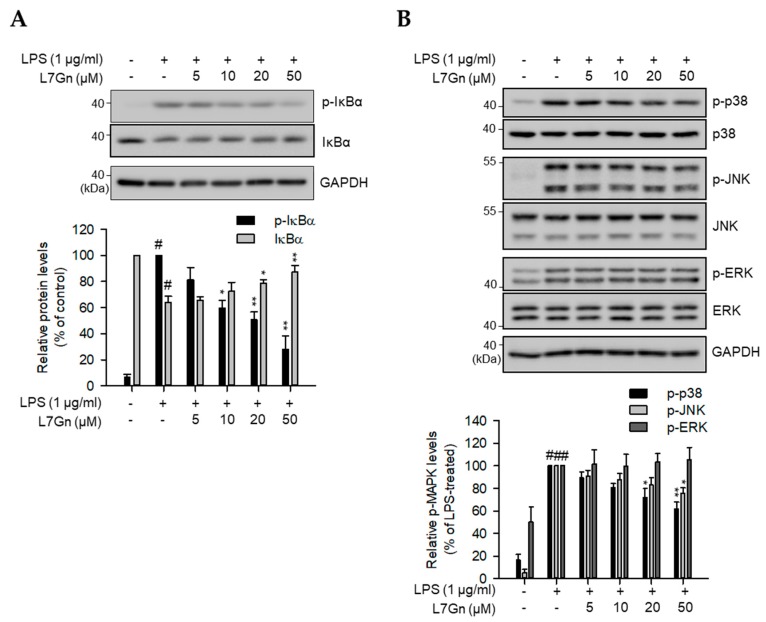
Inhibitory effects of L7Gn on NF-κB and MAPK activation. (**A** and **B**) RAW 264.7 macrophages were pre-treated with different concentrations of L7Gn (0, 5, 10, 20, and 50 μM) for 2 h, followed by LPS (1 μg/mL) stimulation for 3 min (**A**) or 15 min (**B**). Total cell lysates were prepared and immunoblot analysis was performed. The expression of p-IκBα, IκBα (**A**), p-p38, p38, p-JNK, c-Jun N-terminal kinase (JNK), p-ERK, and ERK (**B**) was detected using specific antibodies. Expression levels of p-IκBα and IκBα were normalized to GAPDH levels. Levels of phosphorylated MAPKs were normalized to the corresponding total MAPK levels. Results of the quantitative analyses of phosphorylated or total protein levels after normalization are shown (lower panel). Data represent the mean ± S.D. ^#^ *p* < 0.01 relative to the non-treated control group. * *p* < 0.05 and ** *p* < 0.01 relative to the non-treated (IκBα in **A**) or LPS-treated and 5 μM L7Gn-treated group (**B** and p-IκBα in **A**).

Transcription of inflammatory mediators is chiefly regulated by binding of major transcriptional factors, including NF-κB and AP-1, to the promoter regions of genes encoding these factors [22,23,24]. Considering that IκB degradation and MAPK phosphorylation are the upstream regulatory signaling pathways for the transcriptional activation of NF-κB and AP-1, respectively, immunoblot analyses were performed to detect the inhibitory effect of L7Gn on IκB degradation and MAPK phosphorylation [25,26]. LPS-induced phosphorylation and degradation of IκB were inhibited by L7Gn treatment, suggesting that L7Gn alleviates LPS-induced NF-κB signal activation (Figure 3A). Furthermore, the phosphorylation of p38 and JNK was reduced by L7Gn treatment; however, L7Gn had no effect on ERK phosphorylation (Figure 3B).

It was reported that ERK signaling was not activated by LPS stimulation in Tpl2−/− peritoneal macrophages [27]. However, other major inflammatory signaling pathways, including NF-κB, p38, and JNK, are still activated by LPS. Inactivation of ERK in Tpl2−/− peritoneal macrophages led to low levels of TNF-α production [27]. In this study, L7Gn did not inhibit ERK activation and TNF-α production in LPS-stimulated RAW 264.7 cells, suggesting that moderate inhibition of TNF-α expression by L7Gn might be due to no effect of L7Gn on ERK activation.

### 2.3. L7Gn Suppresses TAK1 Phosphorylation, an Upstream Kinase of NF-κB and MAPKs

**Figure 4:**
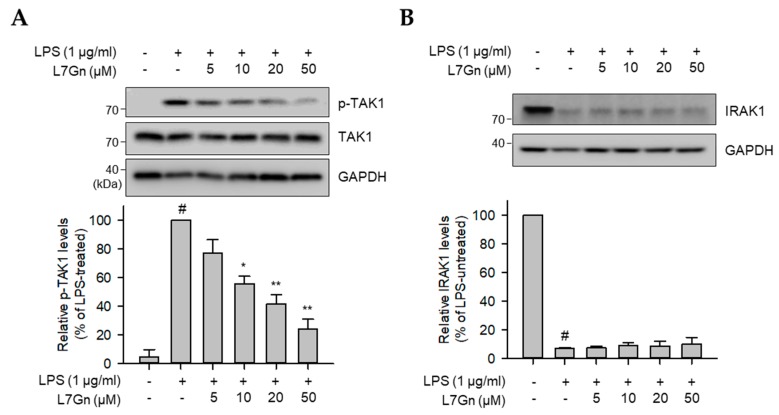
Effects of L7Gn on LPS-induced phosphorylation of TAK1 and degradation of IRAK1. RAW 264.7 cells were pre-treated with L7Gn for 2 h and then treated with LPS (1 μg/mL) for 3 min. Total proteins were subjected to immunoblot analysis using the indicated specific antibodies. (**A**) The level of phosphorylated TAK1 was normalized to that of total TAK1. (**B**) The expression of IRAK1 was normalized to that of GAPDH. Results of the quantitative analyses of phosphorylated or total protein levels after normalization are shown (lower panel). Data represent the mean ± S.D. ^#^ *p* < 0.01 relative to the LPS-untreated control group. ^#^ *p* < 0.01 relative to the LPS-untreated control group. * *p* < 0.05 and ** *p* < 0.01 relative to the LPS-treated and 5 μM L7Gn-treated group.

Previous studies have revealed that NF-κB, p38, and JNK are strongly regulated by an upstream kinase, TAK1 [28]. To validate the action point of L7Gn in LPS-stimulated RAW 264.7 macrophages, the regulatory effect of L7Gn on TAK1 phosphorylation and upstream kinase of TAK1, IRAK1, expression was measured by immunoblot analyses. LPS-induced TAK1 phosphorylation was reduced by L7Gn treatment (Figure 4A). IRAK1 is known to be degraded when it is phosphorylated and, thereby, activated [29]. Thus, an increase in IRAK1 protein expression leads to reduction of phosphorylation and activation of TAK1. However, LPS-induced IRAK1 degradation was not recovered by L7Gn treatment (Figure 4B). This suggests that L7Gn might exert its anti-inflammatory effects in LPS-stimulated macrophages by inhibiting TAK1 phosphorylation, resulting in the inhibition of major inflammatory signaling activators, such as NF-κB, p38, and JNK.

### 2.4. L7Gn Enhances the Expression of Anti-Oxidative Regulators by Activating Nrf2 Expression

**Figure 5:**
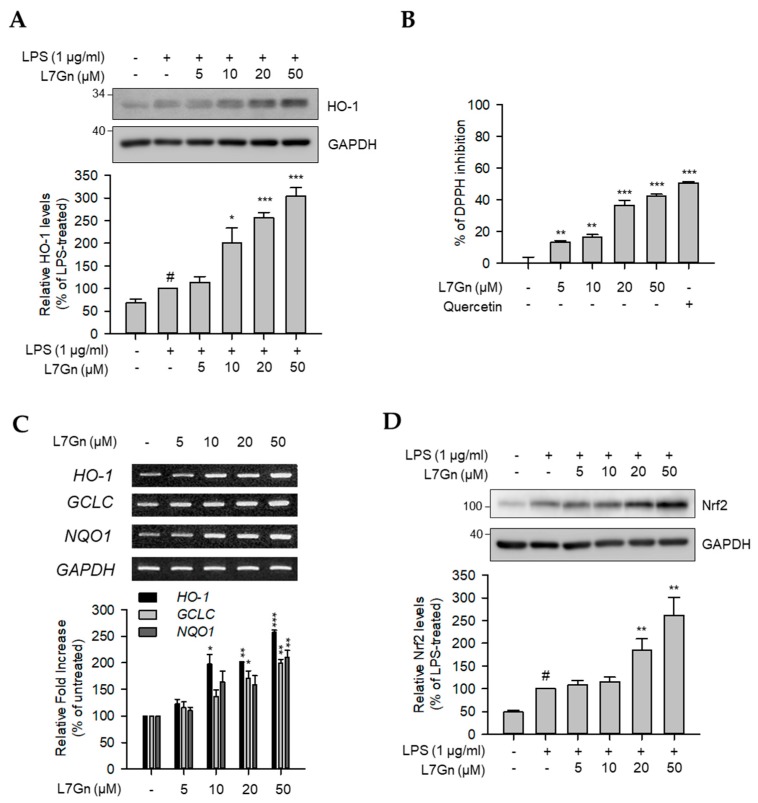
Effects of L7Gn on anti-oxidative regulators. RAW 264.7 cells were pre-treated with L7Gn for 2 h and then treated with LPS (1 μg/mL) for 24 h for immunoblot analyses. (**A**) Total cell lysates were prepared and immunoblot analysis was performed. The expression of HO-1 was detected using specific antibodies. Expression levels of HO-1 were normalized to GAPDH levels. Results of the quantitative analyses of protein levels after normalization are shown (lower panel). Data represent the mean ± S.D. ^#^ *p* < 0.01 relative to the LPS-untreated control group. * *p* < 0.05 and *** *p* < 0.001 relative to the LPS-treated and 5 μM L7Gn-treated group. (**B**) DPPH free radical scavenging activity was represented as the mean ± S.D. ** *p* < 0.01 and *** *p* < 0.001 relative to the non-treated group. (**C**) Total RNA was extracted and reverse transcribed to cDNA. *HO-1*, *GLCL*, and *NQO1* were amplified by PCR and detected on agarose gel. The representative data are shown. * *p* < 0.05, ** *p* < 0.01, and *** *p* < 0.001 relative to the untreated group. (**D**) Total cell lysates were prepared and immunoblot analysis was performed. The expression of Nrf2 was detected using specific antibodies. Expression levels of Nrf2 were normalized to GAPDH levels. Results of the quantitative analyses of protein levels after normalization are shown (lower panel). Data represent the mean ± S.D. ^#^ *p* < 0.01 relative to the LPS-untreated control group. * *p* < 0.05 and ** *p* < 0.01 relative to the LPS-treated and 5 μM L7Gn-treated group.

The effect on HO-1 protein expression by L7Gn treatment was evaluated in LPS-stimulated RAW 264.7 macrophages to determine whether the transcriptional regulation of *iNOS* expression could be due to the enhanced expression of HO-1 as well as the inhibition of major inflammatory signaling pathways. As shown in Figure 5A, HO-1 protein expression was highly induced by L7Gn. Therefore, the anti-oxidative effect of L7Gn based on the engagement of HO-1 in the reduction of oxidative stresses was investigated. Radical scavenging activity was notably induced by L7Gn treatment, indicating that L7Gn has an anti-oxidative property (Figure 5B). Furthermore, the mRNA expression levels of anti-oxidative regulators, including *HO-1*, *GCLC*, and *NQO1*, were measured to clarify the anti-oxidative properties of L7Gn in RAW 264.7 macrophages. As expected, L7Gn increased the mRNA expression levels of *HO-1*, *GCLC*, and *NQO1* (Figure 5C). As these anti-oxidative regulators are controlled by Nrf2, the L7Gn-mediated change in Nrf2 expression was measured by immunoblot analysis, which demonstrated an increase in the Nrf2 protein level by L7Gn treatment (Figure 5D). These data suggest that L7Gn exerts anti-oxidative effects via the Nrf-2-mediated activation of anti-oxidative regulators, such as HO-1, GCLC, and NQO1.

When chemicals are introduced into the human body, they are metabolized by various enzymes, such as oxidases, reductases, and transferases. Although some exceptions do exist, in general, the conjugation of hydrophilic moieties with these chemicals by various transferases through processes, including glucuronidation, sulfonation, and GSH conjugation, leads to the detoxification or inactivation of the toxic or pharmacological effects of the chemicals [30]. Therefore, the comparison of pharmacological or toxic effects between aglycons and their conjugates is a potential strategy to validate whether the metabolic transition of a chemical increases or decreases its pharmacological (or toxic) effects in the body. Luteolin, an aglycon of L7Gn, inhibited over 50% NO production when used at a concentration of 10 µM [18,19]. In this study, L7Gn exhibited 50% NO inhibition at 50 µM, which is a weaker inhibitory effect on NO inhibition than luetolin. Since L7Gn is a metabolite of luteolin [31], this suggests that some of luteolin is metabolized to L7Gn and leads to reduction of anti-inflammatory and anti-oxidative effects in the human body.

## 3. Materials and Methods

### 3.1. Cell Culture and Reagents

The RAW 264.7 macrophage cell line was obtained from ATCC (Manassas, VA, USA). Cells were incubated in Dulbecco’s modified Eagle’s Medium (DMEM; Invitrogen, Carlsbad, CA, USA) supplemented with 10% fetal bovine serum (FBS; Invitrogen) and antibiotics (100 U/mL penicillin and 100 µg/mL streptomycin; GIBCO BRL, Grand Island, NY, USA) at 37 °C in a humidified incubator under 5% CO_2_. L7Gn was purchased from NPBANK (National Institute for Korean Medicine Development, Gyeongsan, Korea). LPS and DMSO were purchased from Sigma-Aldrich (St. Louis, MO, USA). EZ-Cytox solution was from DAEIL lab (Seoul, Korea). TOPscript cDNA synthesis kit and 2X TOPsimple™ DyeMIX-nTaq were purchased from Enzynomics (Daejeon, Korea). Rabbit anti-inhibitor of κBα (IκBα), rabbit anti-phospho (p)-IκBα (Ser-32/36), mouse anti-JNK, mouse anti-p38, rabbit anti-iNOS, goat anti-COX-2, rabbit anti-IL-1 receptor-associated kinase 1 (IRAK1), mouse anti-α-tubulin, and rabbit anti-glyceraldehyde 3-phosphate dehydrogenase (GAPDH) antibodies were purchased from Santa Cruz Biotechnology (Santa Cruz, CA, USA). Rabbit anti-p-p38 (Thr-180/Tyr-182), mouse anti-p-ERK (Thr-202/Tyr-204), rabbit anti-ERK, rabbit anti-p-JNK (Thr-183/Tyr-185), rabbit anti-transforming growth factor-β-activated kinase 1 (TAK1), and rabbit anti-p-TAK1 (Thr-184/Tyr-187) antibodies were purchased from Cell Signaling Technology Inc. (Danvers, MA, USA).

### 3.2. Cell Viability Assay

Cell viability assay was performed as previously described [32]. Briefly, cells were treated with L7Gn for 24 h and, then, 1/20 culture volume of the Ez-Cytox solution was added to each well. Following additional incubation for 1 h, absorbance was measured at 450 nm using a microplate reader (Synergy H1, BioTek Instruments, Inc., Winooski, VT, USA) to validate the viability of cells (absorbance at 650 nm was used as a reference value).

### 3.3. NO Assay

The level of nitrite was measured for determination of NO production in the supernatant of RAW 264.7 cells by the Griess method [33]. Cells were pre-incubated with L7Gn for 2 h and then additionally co-incubated with LPS for 24 h. The NO assay was performed by treating 100 µL of the culture supernatant with 100 µL of Griess reagent in a fresh 96-well plate. A nitrite solution was used to generate a standard curve through serial dilution (1.5625–100 µM). Absorbance was measured at 540 nm using Synergy H1 microplate reader (Biotek) and NO production was calculated by plotting absorbance values on the nitrite standard curve.

### 3.4. RT-PCR

After pre-treatment of RAW 264.7 macrophages with L7Gn for 2 h, the cells were further stimulated with LPS for 3 h. Total RNA was extracted from the cells and subjected to reverse transcription to synthesize complementary DNA, as per the manufacturer’s instructions. The PCR reaction was performed using the primers listed as below: mouse *iNOS* (sense, 5′-ACC AAC TGA CGG GAG ATG AG-3′; antisense, 5′-ATA GCG GAT GAG CTG AGC AT-3′), *COX-2* (sense, 5′-CAA GCA GTG GCA AAG GCC TCC A-3′; antisense, 5′-GGC ACT TGC ATT GAT GGT GGC T-3′), *TNF-α* (sense, 5′-GTG CCA GCC GAT GGG TTG TAC C-3′; antisense, 5′-AGG CCC ACA GTC CAG GTC ACT G-3′), *IL-6* (sense, 5′-TCT TGG GAC TGA TGC TGG TGA C-3′; antisense, 5′-CAT AAC GCA CTA GGT TTG CCG A-3′), *IL-1β* (sense, 5′-AGC TGT GGC AGC TAC CTG TG-3′; antisense, 5′-GCT CTG CTT GTG AGG TGC TG-3′), *HO-1* (sense, 5′-CTG AGG AGC TGC ACC GAA GGG CTG-3′; antisense, 5′-GTG CTT GAC CTC AGG TGT CAT CTC CAG-3′), *NQO1* (sense, 5′-CAT ATG CTG CCA TGT ACG ACA ACG GTC C-3′; antisense, 5′-CTT CCA TCC TTC CAG GAT CTG CAT GCG G-3′), *GCLC* (sense, 5′-GGC ACG GCA TCC TCC AGT TCC TG-3′; antisense, 5′-CAG AAG TAG CCT CCT TCC GGC G-3′), and *GAPDH* (sense, 5′-GTC TTC ACC ACC ATG GAG AAG G-3′; antisense, 5′-CCT GCT TCA CCA CCT TCT TGA T-3′). PCR conditions are as follows; 20–25 cycles of 94 °C (30 s), 60 °C (30 s), and 72 °C (30 s). PCR conditions are as follows; 20–25 cycles of 94 °C (30 s), 60 °C (30 s), and 72 °C (30 s). The resultant amplicons were separated on a 1.5% agarose gel containing EtBr and visualized using a UV transilluminator.

### 3.5. Immunoblot Analysis

Total cell lysates were prepared from cells treated with LPS for indicated times, following pre-treatment with L7Gn for 2 h. Protein samples were prepared for separation by mixing cell lysates with 5× Laemmli sample buffer and, then, boiling the mixture at 100 °C. The samples were then subjected to SDS-PAGE, and the separated proteins were transferred onto nitrocellulose membranes. The membranes were subsequently blocked using 5% non-fat dried milk for 1 h at room temperature (RT) and incubated with primary antibody diluents at 4 °C overnight. Each membrane was subsequently incubated with HRP-conjugated secondary antibody diluents for 1 h at 4 °C and subjected to the detection of desired protein bands using an enhanced chemiluminescence system (Amersham Imager 680, GE Healthcare, Chicago, IL, USA).

### 3.6. Radical Scavenging Assay

The 2,2′-diphenyl-1-picrylhydrazyl (DPPH) free radical scavenging assay was used to measure anti-oxidative activity. L7Gn stock solution (20 mM) was prepared by dissolving L7Gn in 100% DMSO and concentrations of 5, 10, 20 and 50 μM were prepared by diluting the stock solution in 100% MeOH. Then, 100 μL of diluents were transferred to a 96-well plate. Each well then reacted with 100 μL of 0.2 mM DPPH solution. After 30 min incubation at 37 °C, the absorbance at 517 nm was measured, the radical scavenging activity was calculated [(Abs_sample_ − Abs_MeOH_)/Abs_MeOH_] and expressed as relative % value. Quercetin (30 μg/mL, Sigma-Aldrich) was used as the positive control.

### 3.7. Statistical Analysis

The statistical significance of data was analyzed using Student’s t-test. Prism 3.0 (Graphpad software, San Diego, CA, USA) was employed to analyze statistical significances. Results for the experimental replicates (*n* = 9 for the NO assay and cell viability assay / *n* = 3 for PCR and immunoblot analysis) were compared with those for the control group, and *p* < 0.05 was considered as statistically significant. Groups showing statistically significant results are indicated by an asterisk (* *p* < 0.05; **, *p* < 0.01; ***, *p* < 0.001).

## 4. Conclusions

Overall, in this study, the anti-inflammatory and anti-oxidative effects of L7Gn were evaluated in LPS-stimulated RAW 264.7 mouse macrophages. Experimental findings demonstrate that L7Gn exerts anti-inflammatory and anti-oxidative effects by differentially regulating TAK1 and Nrf2, suggesting that L7Gn may be an ideal candidate to alleviate severe inflammatory responses and oxidative stress.
